# 4-{2-[(*E*)-Cyclo­pentyl­idene]hydrazin-1-yl}benzene­sulfonamide

**DOI:** 10.1107/S1600536812012524

**Published:** 2012-03-28

**Authors:** Abdulrahman O. Al-Youbi, Abdullah M. Asiri, Hassan M. Faidallah, Seik Weng Ng, Edward R. T. Tiekink

**Affiliations:** a Chemistry Department, Faculty of Science, King Abdulaziz University, PO Box 80203, Jeddah, Saudi Arabia; b The Center of Excellence for Advanced Materials Research, King Abdulaziz University, Jeddah, PO Box 80203, Saudi Arabia; c Department of Chemistry, University of Malaya, 50603 Kuala Lumpur, Malaysia

## Abstract

The title mol­ecule, C_11_H_15_N_3_O_2_S, features a five-membered ring which is twisted about the middle CH_2_—CH_2_ bond. The benzene ring is inclined with respect to the imine residue [C—N—N—C torsion angle = 165.4 (2)°]. Supra­molecular layers in the *bc* plane are formed by hydrogen bonds between the amine H atoms and sulfonamide O and imine N atoms, as well as by a weak hydrazine H-atom inter­molecular inter­action with the second sulfonamide O atom.

## Related literature

For background to the biological applications of related sulfonamides, see: Al-Saadi *et al.* (2008 ▶). For related structures, see: Asiri *et al.* (2011 ▶, 2012 ▶).
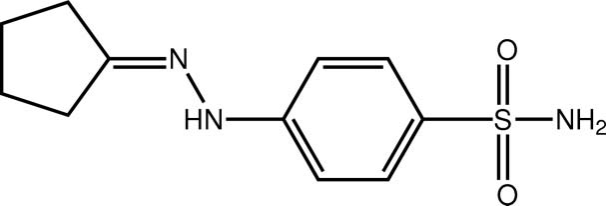


## Experimental

### 

#### Crystal data

C_11_H_15_N_3_O_2_S
*M* *_r_* = 253.32
Monoclinic, 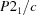

*a* = 14.1173 (14) Å
*b* = 5.2038 (5) Å
*c* = 16.4239 (19) Å
β = 94.019 (10)°
*V* = 1203.6 (2) Å^3^
*Z* = 4
Mo *K*α radiation
μ = 0.26 mm^−1^
*T* = 100 K
0.35 × 0.05 × 0.02 mm

#### Data collection

Agilent SuperNova Dual diffractometer with an Atlas detector
Absorption correction: multi-scan (*CrysAlis PRO*; Agilent, 2011 ▶) *T*_min_ = 0.914, *T*_max_ = 0.995
4782 measured reflections
2768 independent reflections
2007 reflections with *I* > 2σ(*I*)
*R*_int_ = 0.044

#### Refinement

*R*[*F* ^2^ > 2σ(*F* ^2^)] = 0.056
*wR*(*F* ^2^) = 0.123
*S* = 1.03
2768 reflections
166 parameters
3 restraints
H atoms treated by a mixture of independent and constrained refinement
Δρ_max_ = 0.32 e Å^−3^
Δρ_min_ = −0.38 e Å^−3^

### 

Data collection: *CrysAlis PRO* (Agilent, 2011 ▶); cell refinement: *CrysAlis PRO*; data reduction: *CrysAlis PRO*; program(s) used to solve structure: *SHELXS97* (Sheldrick, 2008 ▶); program(s) used to refine structure: *SHELXL97* (Sheldrick, 2008 ▶); molecular graphics: *ORTEP-3* (Farrugia, 1997 ▶) and *DIAMOND* (Brandenburg, 2006 ▶); software used to prepare material for publication: *publCIF* (Westrip, 2010 ▶).

## Supplementary Material

Crystal structure: contains datablock(s) global, I. DOI: 10.1107/S1600536812012524/jj2126sup1.cif

Structure factors: contains datablock(s) I. DOI: 10.1107/S1600536812012524/jj2126Isup2.hkl

Additional supplementary materials:  crystallographic information; 3D view; checkCIF report

## Figures and Tables

**Table 1:** Hydrogen-bond geometry (Å, °)

*D*—H⋯*A*	*D*—H	H⋯*A*	*D*⋯*A*	*D*—H⋯*A*
N1—H1⋯O1^i^	0.88 (1)	2.02 (1)	2.869 (3)	164 (3)
N1—H2⋯N3^ii^	0.88 (1)	2.13 (1)	2.993 (3)	166 (3)
N2—H3⋯O2^iii^	0.88 (1)	2.36 (1)	3.220 (3)	166 (3)

Symmetry codes: (i) 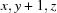; (ii) 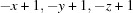; (iii) 
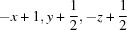
.

**Fig. 1.**
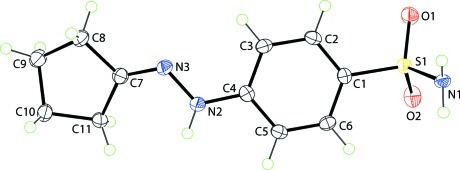
The molecular structure of (I) showing the atom-labelling scheme and displacement ellipsoids at the 50% probability level.

**Fig. 2.**
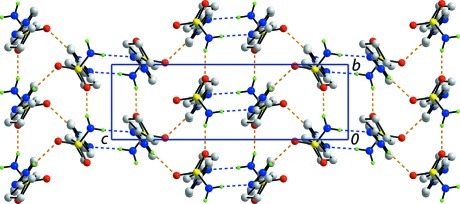
A view of the supramolecular layer in the bc plane in (I). The N—H···O and N—H···N hydrogen bonds are shown as orange and blue dashed lines, respectively.

**Fig. 3.**
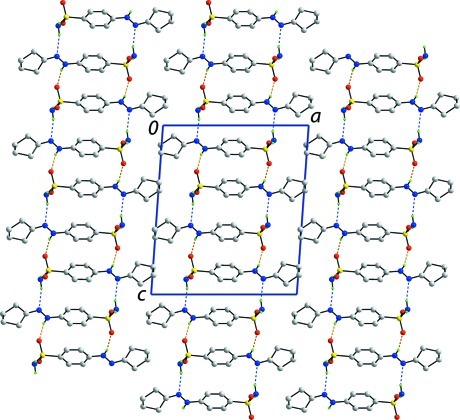
A view in projection down the b axis of the unit-cell contents of (I) showing the stacking of layers along the a axis. The N—H···O and N—H···N interactions are shown as orange and blue dashed lines, respectively.

## supplementary crystallographic information

### Comment

Sulfonamides related to the title compound, 4-(*N*-cyclopentylidenehydrazino)benzensulfonamide (I), have been shown to display biological activity (Al-Saadi *et al.*, 2008). In continuation of structural studies of these sulfonamides (Asiri *et al.*, 2011; Asiri *et al.*, 2012), the crystal and molecular structure of (I) is reported herein.

In (I), Fig. 1, the five-membered ring is twisted about the C9—C10 bond. The imine residue is not co-planar with the benzene ring with a twist apparent about the N2—C4 bond; the C4—N2—N3—C7 torsion angle is 165.4 (2)°.

The crystal packing is dominated by N—H···O and N—H···N hydrogen bonds. The amino-H atoms form hydrogen bonds with a sulfonamide-O and imine-N atoms. The hydrazine-H atom forms a weak intermolecular interaction with the second sulfonamide-O atom, Table 1. The result is the formation of supramolecular layers in the *bc* plane, Fig. 2. Layers stack along the *a* axis with no specific intermolecular interactions between them.

### Experimental

Cyclopentanone (0.84 g, 10 mmol) in ethanol was refluxed with *p*-sulfamylphenylhydrazine (2.2 g, 10 mmol) for 1 h. The reaction mixture was cooled and the precipitated product was collected by filtration, washed with ethanol, dried and recrystallized from its ethanol solution. Yield: 78%. *M*. pt: 475–477 K.

### Refinement

Carbon-bound H-atoms were placed in calculated positions [C—H = 0.95 to 0.99 Å, *U*_iso_(H) = 1.2*U*_eq_(C)] and were included in the refinement in the riding model approximation. The N-bound H-atoms were located in a difference Fourier map and were refined with a distance restraint of N—H = 0.88±0.01 Å; their *U*_iso_ values were refined.

### Crystal data

**Table:**

C_11_H_15_N_3_O_2_S	*F*(000) = 536
*M**_r_* = 253.32	*D*_x_ = 1.398 Mg m^−^^3^
Monoclinic, *P*2_1_/*c*	Mo *K*α radiation, λ = 0.71073 Å
Hall symbol: -P 2ybc	Cell parameters from 1078 reflections
*a* = 14.1173 (14) Å	θ = 2.5–27.5°
*b* = 5.2038 (5) Å	µ = 0.26 mm^−^^1^
*c* = 16.4239 (19) Å	*T* = 100 K
β = 94.019 (10)°	Plate, colourless
*V* = 1203.6 (2) Å^3^	0.35 × 0.05 × 0.02 mm
*Z* = 4	

### Data collection

**Table:**

Agilent SuperNova Dual diffractometer with an Atlas detector	2768 independent reflections
Radiation source: SuperNova (Mo) X-ray Source	2007 reflections with *I* > 2σ(*I*)
Mirror monochromator	*R*_int_ = 0.044
Detector resolution: 10.4041 pixels mm^-1^	θ_max_ = 27.6°, θ_min_ = 2.5°
ω scan	*h* = −18→11
Absorption correction: multi-scan (*CrysAlis PRO*; Agilent, 2011)	*k* = −4→6
*T*_min_ = 0.914, *T*_max_ = 0.995	*l* = −21→21
4782 measured reflections	

### Refinement

**Table:**

Refinement on *F*^2^	Primary atom site location: structure-invariant direct methods
Least-squares matrix: full	Secondary atom site location: difference Fourier map
*R*[*F*^2^ > 2σ(*F*^2^)] = 0.056	Hydrogen site location: inferred from neighbouring sites
*wR*(*F*^2^) = 0.123	H atoms treated by a mixture of independent and constrained refinement
*S* = 1.03	*w* = 1/[σ^2^(*F*_o_^2^) + (0.0372*P*)^2^ + 0.8414*P*] where *P* = (*F*_o_^2^ + 2*F*_c_^2^)/3
2768 reflections	(Δ/σ)_max_ = 0.001
166 parameters	Δρ_max_ = 0.32 e Å^−^^3^
3 restraints	Δρ_min_ = −0.38 e Å^−^^3^

### Fractional atomic coordinates and isotropic or equivalent isotropic displacement parameters (Å^2^)

**Table:**

	*x*	*y*	*z*	*U*_iso_*/*U*_eq_	
S1	0.28398 (5)	0.43647 (12)	0.35890 (4)	0.01924 (18)	
O1	0.26446 (13)	0.1919 (3)	0.39559 (13)	0.0304 (5)	
O2	0.25331 (13)	0.4780 (4)	0.27417 (11)	0.0276 (5)	
N1	0.23242 (16)	0.6501 (4)	0.41081 (14)	0.0185 (5)	
H1	0.238 (2)	0.811 (2)	0.3960 (17)	0.028 (8)*	
H2	0.244 (2)	0.626 (6)	0.4636 (7)	0.042 (10)*	
N2	0.70084 (16)	0.5585 (5)	0.36168 (14)	0.0238 (5)	
H3	0.7234 (19)	0.672 (4)	0.3286 (14)	0.025 (8)*	
N3	0.76230 (15)	0.4003 (4)	0.40739 (13)	0.0210 (5)	
C1	0.40782 (17)	0.4817 (5)	0.36814 (15)	0.0172 (5)	
C2	0.46722 (18)	0.3075 (5)	0.41081 (15)	0.0192 (6)	
H2A	0.4406	0.1709	0.4401	0.023*	
C3	0.56513 (18)	0.3314 (5)	0.41102 (16)	0.0199 (6)	
H3A	0.6054	0.2114	0.4400	0.024*	
C4	0.60411 (18)	0.5343 (5)	0.36816 (15)	0.0182 (5)	
C5	0.54389 (18)	0.7151 (5)	0.32815 (16)	0.0193 (6)	
H5	0.5703	0.8571	0.3012	0.023*	
C6	0.44676 (18)	0.6888 (5)	0.32749 (15)	0.0187 (6)	
H6	0.4063	0.8108	0.2996	0.022*	
C7	0.84829 (18)	0.3891 (5)	0.38745 (16)	0.0202 (6)	
C8	0.92115 (18)	0.2283 (6)	0.43640 (17)	0.0247 (6)	
H8A	0.8971	0.0522	0.4445	0.030*	
H8B	0.9378	0.3070	0.4904	0.030*	
C9	1.00726 (19)	0.2246 (5)	0.38442 (17)	0.0252 (6)	
H9A	1.0673	0.2150	0.4193	0.030*	
H9B	1.0041	0.0767	0.3464	0.030*	
C10	1.00007 (18)	0.4790 (5)	0.33771 (17)	0.0248 (6)	
H10A	1.0339	0.4684	0.2870	0.030*	
H10B	1.0275	0.6213	0.3717	0.030*	
C11	0.89299 (18)	0.5202 (5)	0.31788 (16)	0.0220 (6)	
H11A	0.8771	0.7055	0.3160	0.026*	
H11B	0.8718	0.4405	0.2650	0.026*	

### Atomic displacement parameters (Å^2^)

**Table:**

	*U*^11^	*U*^22^	*U*^33^	*U*^12^	*U*^13^	*U*^23^
S1	0.0185 (3)	0.0147 (3)	0.0246 (4)	−0.0019 (3)	0.0025 (3)	−0.0034 (3)
O1	0.0247 (11)	0.0141 (9)	0.0531 (15)	−0.0038 (8)	0.0079 (10)	−0.0002 (9)
O2	0.0233 (10)	0.0373 (12)	0.0215 (10)	−0.0024 (9)	−0.0031 (8)	−0.0091 (9)
N1	0.0220 (12)	0.0134 (11)	0.0203 (13)	0.0014 (9)	0.0032 (10)	0.0016 (10)
N2	0.0204 (12)	0.0261 (13)	0.0255 (13)	0.0024 (11)	0.0054 (9)	0.0109 (11)
N3	0.0200 (12)	0.0238 (12)	0.0191 (12)	0.0026 (10)	0.0003 (9)	0.0055 (10)
C1	0.0169 (13)	0.0186 (13)	0.0162 (13)	−0.0002 (10)	0.0021 (10)	−0.0031 (10)
C2	0.0242 (14)	0.0160 (13)	0.0180 (14)	−0.0013 (11)	0.0063 (11)	0.0008 (11)
C3	0.0232 (14)	0.0176 (13)	0.0189 (14)	0.0024 (11)	0.0014 (11)	0.0027 (11)
C4	0.0187 (13)	0.0201 (13)	0.0162 (13)	−0.0010 (11)	0.0029 (10)	−0.0026 (11)
C5	0.0229 (14)	0.0166 (13)	0.0189 (14)	−0.0014 (11)	0.0050 (11)	0.0004 (11)
C6	0.0230 (14)	0.0154 (12)	0.0175 (13)	0.0016 (11)	0.0001 (10)	−0.0009 (11)
C7	0.0201 (14)	0.0214 (14)	0.0190 (14)	−0.0013 (11)	−0.0006 (10)	0.0003 (11)
C8	0.0210 (14)	0.0310 (15)	0.0218 (15)	0.0034 (12)	−0.0003 (11)	0.0083 (12)
C9	0.0239 (15)	0.0278 (15)	0.0237 (15)	0.0043 (12)	0.0009 (11)	0.0027 (12)
C10	0.0179 (14)	0.0315 (16)	0.0248 (15)	0.0021 (12)	0.0003 (11)	0.0053 (12)
C11	0.0191 (14)	0.0257 (15)	0.0211 (14)	−0.0011 (11)	0.0009 (11)	0.0058 (12)

### Geometric parameters (Å, º)

**Table:**

S1—O2	1.4444 (19)	C5—C6	1.377 (4)
S1—O1	1.4430 (19)	C5—H5	0.9500
S1—N1	1.606 (2)	C6—H6	0.9500
S1—C1	1.760 (3)	C7—C8	1.513 (4)
N1—H1	0.878 (10)	C7—C11	1.507 (3)
N1—H2	0.879 (10)	C8—C9	1.534 (4)
N2—N3	1.379 (3)	C8—H8A	0.9900
N2—C4	1.383 (3)	C8—H8B	0.9900
N2—H3	0.875 (10)	C9—C10	1.530 (4)
N3—C7	1.281 (3)	C9—H9A	0.9900
C1—C2	1.391 (4)	C9—H9B	0.9900
C1—C6	1.400 (4)	C10—C11	1.539 (3)
C2—C3	1.388 (4)	C10—H10A	0.9900
C2—H2A	0.9500	C10—H10B	0.9900
C3—C4	1.403 (4)	C11—H11A	0.9900
C3—H3A	0.9500	C11—H11B	0.9900
C4—C5	1.401 (4)		
			
O2—S1—O1	118.77 (12)	C5—C6—H6	120.1
O2—S1—N1	106.95 (12)	C1—C6—H6	120.1
O1—S1—N1	106.37 (12)	N3—C7—C8	120.7 (2)
O2—S1—C1	106.97 (12)	N3—C7—C11	128.9 (2)
O1—S1—C1	107.43 (12)	C8—C7—C11	110.4 (2)
N1—S1—C1	110.25 (12)	C7—C8—C9	104.3 (2)
S1—N1—H1	117.4 (19)	C7—C8—H8A	110.9
S1—N1—H2	111 (2)	C9—C8—H8A	110.9
H1—N1—H2	113 (3)	C7—C8—H8B	110.9
N3—N2—C4	119.4 (2)	C9—C8—H8B	110.9
N3—N2—H3	119.8 (19)	H8A—C8—H8B	108.9
C4—N2—H3	120.8 (19)	C10—C9—C8	103.9 (2)
C7—N3—N2	117.3 (2)	C10—C9—H9A	111.0
C2—C1—C6	119.9 (2)	C8—C9—H9A	111.0
C2—C1—S1	121.1 (2)	C10—C9—H9B	111.0
C6—C1—S1	118.9 (2)	C8—C9—H9B	111.0
C3—C2—C1	120.5 (2)	H9A—C9—H9B	109.0
C3—C2—H2A	119.7	C9—C10—C11	104.9 (2)
C1—C2—H2A	119.7	C9—C10—H10A	110.8
C2—C3—C4	119.5 (2)	C11—C10—H10A	110.8
C2—C3—H3A	120.3	C9—C10—H10B	110.8
C4—C3—H3A	120.3	C11—C10—H10B	110.8
N2—C4—C5	118.2 (2)	H10A—C10—H10B	108.9
N2—C4—C3	122.1 (2)	C7—C11—C10	103.5 (2)
C5—C4—C3	119.6 (2)	C7—C11—H11A	111.1
C6—C5—C4	120.6 (2)	C10—C11—H11A	111.1
C6—C5—H5	119.7	C7—C11—H11B	111.1
C4—C5—H5	119.7	C10—C11—H11B	111.1
C5—C6—C1	119.8 (2)	H11A—C11—H11B	109.0
			
C4—N2—N3—C7	165.4 (2)	N2—C4—C5—C6	175.1 (2)
O2—S1—C1—C2	−133.3 (2)	C3—C4—C5—C6	−2.9 (4)
O1—S1—C1—C2	−4.8 (2)	C4—C5—C6—C1	0.9 (4)
N1—S1—C1—C2	110.7 (2)	C2—C1—C6—C5	1.7 (4)
O2—S1—C1—C6	42.8 (2)	S1—C1—C6—C5	−174.46 (19)
O1—S1—C1—C6	171.4 (2)	N2—N3—C7—C8	177.6 (2)
N1—S1—C1—C6	−73.1 (2)	N2—N3—C7—C11	−2.2 (4)
C6—C1—C2—C3	−2.4 (4)	N3—C7—C8—C9	169.5 (3)
S1—C1—C2—C3	173.71 (19)	C11—C7—C8—C9	−10.6 (3)
C1—C2—C3—C4	0.4 (4)	C7—C8—C9—C10	28.9 (3)
N3—N2—C4—C5	173.1 (2)	C8—C9—C10—C11	−36.8 (3)
N3—N2—C4—C3	−8.9 (4)	N3—C7—C11—C10	168.0 (3)
C2—C3—C4—N2	−175.7 (2)	C8—C7—C11—C10	−11.8 (3)
C2—C3—C4—C5	2.3 (4)	C9—C10—C11—C7	29.8 (3)

### Hydrogen-bond geometry (Å, º)

**Table:**

*D*—H···*A*	*D*—H	H···*A*	*D*···*A*	*D*—H···*A*
N1—H1···O1^i^	0.88 (1)	2.02 (1)	2.869 (3)	164 (3)
N1—H2···N3^ii^	0.88 (1)	2.13 (1)	2.993 (3)	166 (3)
N2—H3···O2^iii^	0.88 (1)	2.36 (1)	3.220 (3)	166 (3)

Symmetry codes: (i) *x*, *y*+1, *z*; (ii) −*x*+1, −*y*+1, −*z*+1; (iii) −*x*+1, *y*+1/2, −*z*+1/2.
